# Accumulating evidence from meta-analyses of prognostic studies on oral cancer: towards biomarker-driven patient selection

**DOI:** 10.1186/s12885-024-13317-z

**Published:** 2024-12-18

**Authors:** Alhadi Almangush, Rasheed Omobolaji Alabi, Matti Pirinen, Antti Mäkitie, Ilmo Leivo

**Affiliations:** 1 Department of Pathology, University of Helsinki, Haartmaninkatu 3, Helsinki, FIN-00014 P.O. Box 21, Finland; 2 Institute of Biomedicine, Pathology, University of Turku, Kiinamyllynkatu 10 D 5035, Turku, 20520 Finland; 3 Research Program in Systems Oncology, Faculty of Medicine, University of Helsinki, Helsinki, Finland; 4 Faculty of Dentistry, Misurata University, Misurata, Libya; 5 Institute for Molecular Medicine Finland (FIMM), Helsinki Institute of Life Science (HiLIFE), University of Helsinki, Helsinki, Finland; 6 Department of Public Health, University of Helsinki, Helsinki, Finland; 7 Department of Mathematics and Statistics, University of Helsinki, Helsinki, Finland; 8 Department of Otorhinolaryngology - Head and Neck Surgery, University of Helsinki and Helsinki University Hospital, P.O. Box 263, Helsinki, FI-00029 HUS Finland; 9 Division of Ear, Nose and Throat Diseases, Department of Clinical Sciences, Intervention and Technology, Karolinska Institutet and Karolinska University Hospital, Stockholm, Sweden

**Keywords:** Oral cancer, Oral squamous cell carcinoma, Histopathologic markers, Meta-analysis, Prognosis

## Abstract

**Background:**

Many histopathologic prognostic markers, identified by routine hematoxylin and eosin (HE) staining, have been proposed for predicting the survival of patients with oral squamous cell carcinoma (OSCC). Subsequently, several meta-analyses have been conducted on these prognostic markers. We sought to analyze the accumulated evidence from these meta-analyses.

**Methods:**

An electronic database search of PubMed, Scopus, Ovid Medline, Web of Science, and Cochrane Library was conducted to retrieve all meta-analysis articles published on histopathologic prognostic markers of OSCC. The risk of bias of the included studies was analyzed using the Risk of Bias in Systematic Reviews (ROBIS) tool. The synthesis of the results was conducted following the guidelines of Preferred Reporting Items for Systematic Reviews and Meta-Analyses (PRISMA).

**Results:**

There were 16 meta-analysis articles published on the histological prognostic markers of OSSC. The accumulated evidence from these meta-analyses highlighted the powerful prognostic value of depth of invasion, tumor thickness, perineural invasion, lymphovascular invasion, worst pattern of invasion, tumor budding, and tumor-stroma ratio. The highest odds ratio (OR) of a relationship between a histopathologic prognostic marker and outcome was for the depth of invasion (OR 10.16, 95% CI 5.05–20.46) and tumor thickness (OR 7.32, 95% CI 5.3–10.1) in predicting lymph node metastasis.

**Conclusion:**

The published meta-analyses present robust evidence on the significance of emerging histopathologic markers, namely, worst pattern of invasion, tumor budding, and tumor-stroma ratio. It is time to consider such markers in daily pathology reporting and risk stratification of OSCC.

**Supplementary Information:**

The online version contains supplementary material available at 10.1186/s12885-024-13317-z.

## Background

Oral squamous cell carcinoma (OSCC) is the most common cancer of the oral cavity with an increasing incidence in many countries [1]. The treatment of choice of OSCC is surgery with adjuvant (chemo)radiotherapy. Most recently, immunotherapy was introduced for selected cases [2]. In daily practice, OSCC patients’ selection relies on the TNM staging system which in common experience may sometimes not reveal the biological behavior of the tumor. Although the survival rate of patients with OSCC has improved, still many patients get relapses of their tumor and die from the disease even when diagnosed at an early stage (T1-T2N0M0) [3–5]. Therefore, there are continuous research efforts to identify prognostic biomarkers that could accurately predict the behavior of OSCC.

Histopathologic assessment of OSCC using hematoxylin and eosin (HE) staining is the cornerstone of routine pathology practice. In addition to diagnostic assessment, a prognostic assessment by evaluation of clinically relevant histopathologic features has been introduced [6]. The latter has received attention as it is simple, cost-effective, and can be performed during the first assessment of the tumor sample, i.e., without requesting any additional staining. In clinical decision making, only a few histopathologic parameters are considered (e.g., depth of invasion). Of note, evidence from recent research emphasizes the prognostic power of new histologic features including tumor budding, tumor-stroma ratio and tumor-infiltrating lymphocytes [7–9]. Towards a personalized treatment of OSCC, multiple prognostic markers are necessary preferably assessing characteristics of both the tumor and its surrounding stromal microenvironment. Of note, a recent study has added tumor-stroma ratio to the TNM staging system to improve outcome prognostication in oral tongue cancer [10]. Furthermore, recent studies have introduced a tumor-microenvironment grading system combining tumor-stroma ratio and a score of stromal tumor-infiltrating lymphocytes (TILs) [11, 12].

However, the path in development of clinically reliable prognostic markers remains challenging. Important steps in considering a newly introduced marker for clinical implementation include validation by independent studies. Meta-analysis is an important tool in evidence-based medicine allowing for the analysis of accumulated evidence when drawing definitive conclusions from multiple studies [13]. In this article, we have summarized the accumulated evidence from the meta-analyses so far conducted on histologic markers assessed using HE-stained sections of OSCC. Our summary of published meta-analyses will be useful towards evidence-based clinical decision-making as it gathers the accumulated evidence into one article.

## Methods

We conducted a systematic search of Web of Science, Scopus, Ovid Medline, and PubMed from inception until October 2024. The following keywords were used in the search strategy: “Oral cancer” AND “Marker” AND “Meta-analysis” as presented in Supplementary Table 1. This systematic review was conducted following the Preferred Reporting Items for Systematic Reviews and Meta-Analyses (PRISMA) guidelines. The PRISMA checklist for the Abstract and the meta-analysis [14] is presented in Supplementary Tables 2 and 3. This systematic review is registered in PROSPERO (ID: CRD42024579935). Two authors (AA & RA) performed the search and selected the relevant articles. The interobserver reliability between these authors was measured using Cohen’s Kappa coefficient (κ = 0.92). In case of disagreement about the inclusion of an article, a consensus discussion was conducted to reach an agreement. The references of the relevant articles were also checked.

### Research question

Our paper focused on this research question: What are the histologic markers that are validated by meta-analysis in OSCC and what are the findings of these meta-analyses?

### Inclusion criteria

The search strategy was restricted to articles in English. The inclusion criterion was meta-analysis on prognostic value of histologic marker/s derived from HE-stained sections of OSCC. All prognostic outcomes included in the relevant studies (Table 1) were considered. These included overall survival, disease-specific survival, disease-free survival, and lymph node metastasis.

**Table 1:** Summary of meta-analysis articles published on the histopathologic prognostic markers of oral squamous cell carcinoma

**Article number**	Authors (Year)	**Journal**	Title of the study	**Site**	Marker/s included in the meta-analysis	no. of studies	Outcome	Prognostic value from each meta-analysis	Conclusion of each meta-analysis
						(no. of cases)		HR, OR (95%CI)	
**1**	Huang SH et al. (2009)	*Cancer*	Predictive value of tumor thickness for cervical lymph-node involvement in squamous cell carcinoma of the oral cavity	Oral cavity	Tumor thickness	16 studies	LNM	OR 7.32 (5.3–10.1)	Tumor thickness is a powerful prognostic marker for lymph node involvement (cutoff point = 4 mm).
						(1136 patients)			
**2**	Almangush A et al. (2018)	*Br J Cancer*	Tumor budding in oral squamous cell carcinoma: a meta-analysis	Oral cavity	Tumor budding	4 studies	LNM	OR 3.74 (2.52–5.55)	Tumor budding is a simple marker that has a prominent prognostic value for OSCC.
						(653 patients)			
						3 studies	DFS	HR 1.83 (1.34–2.50)	
						(543 patients)			
						3 studies	OS	HR 1.78 (1.33–2.38)	
						(643 patients)			
**3**	Zhu Y et al. (2019)	*Head & Neck*	Impact of tumor budding in head and neck squamous cell	Oral cavity	Tumor budding	3 studies	OS	HR 1.94 (1.30–2.89)	Tumor budding associated with poor survival in cT1-2N0 OSCC.
			carcinoma: A meta-analysis*			(475 patients)		*P* = 0.001	
**4**	Zhu J et al. (2019)	*Acta Otolaryngol*	Perineural invasion as a prognostic factor in head and neck squamous cell carcinoma: a systematic review and meta-analysis*	Oral cavity	Perineural invasion	(1451 patients)	OS	HR 2.36 (1.56–3.56)	Perineural invasion associated with OS of oral cancer patients.
								*P* = 0.002	
**5**	Karjol U et al. (2020)	*Cureus*	Prognostic Role of Tumor Budding in Carcinoma Tongue	Tongue	Tumor budding	7 studies	LNM	HR 3.07 (2.08–4.52)	Tumor budding is a valuable predictor of LNM and OS in TSCC and should be considered in a staging system.
						(907 patients)		*P* ≤ 0.00001	
						5 studies	OS	HR 2.40 (1.84–3.14)	
						(891 patients)		*P* ≤ 0.00001	
**6**	Caldeira PC et al. (2020)	*Oral Dis*	Tumor depth of invasion and prognosis of early-stage OSCC: A meta-analysis	Oral cavity	Depth of invasion	6 studies	LNM	OR 10.16 (5.05–20.46)	Depth of invasion is a valuable prognostic marker for early-stage OSCC.
						(416 patients)			
						2 studies	Recurrence	OR 3.83 (1.60–9.14)	
						(146 patients)			
**7**	Choudhary N et al. (2021)	*J Oral Biol Craniofac Res.*	Tumor associated tissue eosinophilia in oral squamous cell carcinoma: A systematic review and meta-analysis	Oral cavity	Tumor associated tissue eosinophilia	3 studies	OS	HR 0.45 (0.30–0.65)	Tumor associated tissue eosinophilia is an important prognostic marker in OSCC.
						(311 patients)		*P* < 0.0001	
						4 studies	DFS	HR 2.33 (0.74–7.37)	
						(284 patients)			
**8**	Huang S et al. (2021)	*Oral Surg Oral Med Oral Pathol Oral Radiol*	Impact of lymphovascular invasion in oral squamous cell carcinoma: A meta-analysis	Oral cavity	Lymphovascular invasion	18 studies	LNM	OR 5.34 (3.44–8.30)	Lymphovascular invasion associates with LNM and predicts OSCC with poor survival.
						(2161 patients)		*P* < 0.00001	
						11 studies	OS	HR 1.55 (1.42–1.69)	
						(12783 patients)		*P* < 0.00001	
						7 studies	DSS	HR 1.76 (1.48–2.09)	
						(3470 patients)		*P* < 0.00001	
						3 studies	DFS	HR 1.20 (0.89–1.62)	
						(731 patients)		*P* = 0.24	
**9**	Almangush A et al. (2021)	*BMC Cancer*	Clinical significance of tumor-stroma ratio in head and neck cancer: a systematic review and meta-analysis*	Oral cavity	Tumor-stroma ratio	3 studies	DSS	HR 2.10 (1.56–2.84)	Evaluation of tumor-stroma ratio has a promising prognostic impact and can be incorporated in routine pathology.
						(776 patients)			
						3 studies	DFS	HR 1.84 (1.38–2.46)	
						(776 patients)			
**10**	Li J et al. (2021)	*Front Oncol*	Prognostic value of perineural invasion in oral tongue squamous	Oral tongue	Perineural invasion	3 studies	Recurrence	HR 1.726 (1.070–2.786) *P* = 0.025	Perineural invasion associated significantly with recurrence and
			cell carcinoma: a systematic review and meta-analysis			(749 patients)			survival of OTSCC.
						5 studies	OS	HR 1.944 (1.387–2.724)	
						(1094 patients)		*P* < 0.001	
						7 studies	DFS	HR 2.128 (1.532–2.955)	
						(1597 patients)		*P* < 0.001	
						5 studies	DSS	HR 1.927 (1.402–2.650)	
						(1376 patients)		*P* < 0.001	
**11**	Dolens EDS et al. (2021)	*Front Oncol*	The impact of histopathological features on the prognosis of oral	Oral cavity	Depth of invasion	27 studies	OS	HR 1.94 (1.54–2.44)	The meta-analyses have confirmed the prognostic impact of depth of invasion, lymphovascular invasion, perineural invasion, status of surgical margin, tumor thickness and pattern of invasion. They have also highlighted the clinical significance of newly introduced markers, namely, tumor budding and tumor-stroma ratio in OSCC.
			squamous cell carcinoma: a comprehensive review and meta-analysis			(7324 patients)		*P* < 0.00001	
						11 studies	DSS	HR 1.45 (1.29–1.64)	
						(7781 patients)		*P* < 0.00001	
						27 studies	DFS	HR 1.53 (1.29–1.81)	
						(6348 patients)		*P* < 0.00001	
					Perineural invasion	33 studies (10045 patients)	OS	HR 1.66 (1.51–1.82)	
								*P* < 0.00001	
						26 studies	DSS	HR 1.63 (1.46–1.83)	
						(7523 patients)		*P* < 0.00001	
						45 studies (15268 patients)	DFS	HR 1.62 (1.49–1.76)	
								*P* < 0.00001	
					Lymphovascular invasion	30 studies (30481 patients)	OS	HR 1.81 (1.55–2.13)	
								*P* < 0.00001	
						13 studies	DSS	HR 1.71 (1.46–2.01)	
						(4411 patients)		*P* < 0.00001	
						30 studies	DFS	HR 1.56 (1.22–1.99)	
						(8187 patients)		*P* = 0.0003	
					Surgical margins	31 studies (63470 patients)	OS	HR 1.56 (1.41–1.73)	
								*P* < 0.00001	
						19 studies (20680 patients)	DSS	HR 1.71 (1.58–1.85)	
								*P* < 0.00001	
						25 studies (15300 patients)	DFS	HR 2.47 (1.90–3.21)	
								*P* < 0.00001	
					Tumor thickness	5 studies	OS	HR 1.09 (1.02–1.16)	
						(1651 patients)		*P* = 0.01	
						3 studies	DSS	HR 1.07 (1.01–1.13)	
						(638 patients)		*P* = 0.02	
						4 studies	DFS	HR 2.22 (1.43–3.45)	
						(1556 patients)		*P* = 0.0004	
					Cohesive system	4 studies	OS	HR 2.25 (1.56–3.23)	
						(543 patients)		*P* < 0.0001	
						4 studies	DSS	HR 2.63 (1.56–4.46)	
						(1229 patients)		*P* = 0.0003	
						4 studies	DFS	HR 2.20 (1.37–3.63)	
						(505 patients)		*P* = 0.001	
					Worst pattern of invasion	2 studies	OS	HR 2.40 (1.19–4.84)	
						(420 patients)		*P* = 0.01	
						2 studies	DSS	HR 2.42 (1.00-5.88)	
						(122 patients)		*P* = 0.05	
						5 studies	DFS	HR 2.82 (2.03–3.91)	
						(892 patients)		*P* < 0.00001	
					Tumor budding	5 studies	OS	HR 2.96 (1.36–6.45)	
						(986 patients)		*P* = 0.006	
						5 studies	DSS	HR 1.72 (1.35–2.18)	
						(969 patients)		*P* < 0.00001	
						5 studies	DFS	HR 2.02 (1.50–2.71)	
						(1142 patients)		*P* < 0.00001	
					Tumor-stroma ratio	1 study	OS	HR 1.69 (1.02–2.81)	
						(226 patients)		*P* = 0.04	
						3 studies	DSS	HR 2.26 (1.65–3.11)	
						(724 patients)		*P* < 0.00001	
						4 studies	DFS	HR 2.05 (1.59–2.64)	
						(950 patients)		*P* < 0.00001	
**12**	Wahab A et al. (2022)	*Oral Dis*	The budding and depth of invasion model in oral cancer:	Oral cavity	Combined score of tumor budding and depth of invasion (i.e. BD model)	3 studies	DFS	HR 2.02 (1.44–2.85)	The BD model is a reliable prognosticator in OSCC and can be considered for treatment planning of OSCC.
			A systematic review and meta-analysis			(486 studies)			
**13**	Elseragy A et al. (2022)	*Head & Neck*	Emerging histopathologic markers in early-stage oral tongue cancer: A systematic review and meta-analysis	Oral tongue	Tumor budding	4 studies	OS	HR 2.32 (1.40–3.84)	Tumor budding, tumor-stroma ratio and worst pattern of invasion associate significantly with the prognosis of early oral tongue cancer.
						(942 patients)		*P* < 0.01	
						2 studies	DSS	HR 1.89 (1.13–3.15)	
						(461 patients)		*P* = 0.02	
					Tumor-stroma ratio	2 studies	DFS	HR 1.75 (1.24–2.48)	
						(522 patients)		*P* < 0.01	
						2 studies	DSS	HR 1.69 (1.19–2.42)	
						(522 patients)		*P* < 0.01	
					Worst pattern of invasion	3 studies	DFS	HR 1.95 (1.04–3.64)	
						(567 patients)		*P* = 0.04	
**14**	Feitosa SG et al. (2023)	*Asian Pac J Cancer Prev*	Tumor budding and poor prognosis in oral cancer: a systematic review and meta-analysis	Oral cavity	Tumor budding	8 studies	OS	HR 3.11 (2.06–4.69)	Tumor budding is a reliable prognostic marker for oral cancer.
						(1888 patients)		*P* < 0.01	
						9 studies	DSS	HR 2.43 (1.94–3.03)	
						(1308 patients)		*P* < 0.01	
						12 studies	DFS	HR 1.94 (1.44–2.62)	
						(2130 patients)		*P* < 0.01	
**15**	Silva FFVE et al. (2024)	*Crit Rev Oncol Hematol*	Tumor budding is a prognostic factor in head and neck squamous cell	Oral cavity	Tumor budding	7 studies	LNM	OR 4.48 (2.97–6.76)	Tumor budding is associated with a worse survival.
			carcinoma: A comprehensive meta-analysis and trial sequential analysis*			(976 patients)			
**16**	Panchannavar GS et al. (2024)	*Oral Biol Craniofac Res*	Tumor budding is a prognostic marker for overall survival and not for lymph node metastasis in Oral Squamous Cell Carcinoma - Systematic Review Update and Meta-Analysis	Oral cavity	Tumor budding	9 studies	LNM	OR 2.10 (0.00-4.20)	Tumor budding is associated with OS in OSCC, but not with LNM.
						(1381 patients)			
						11 studies	OS	HR 2.29 (1.81–2.76)	
						(2211 patients)			

**Notes**: *Star on the title of the study indicating meta-analysis articles designed for head and neck cancer but provided separate meta-analysis for oral squamous cell carcinoma

**Cohesive mode**: Sheets or strands with > 15 cells. **Non cohesive mode**: Narrow strands or non-cohesive small groups (< 15 cells) or single cells

**Abbreviations**: BD: Budding and depth of invasion; CI: confidence interval; DFS: disease-free survival; DSS: disease-specific survival; HR: hazard ratio; LNM: lymph node metastasis; OR: odds ratio; OS: overall survival; OSCC: Oral squamous cell carcinoma

### Exclusion criteria

**Fig. 1:**
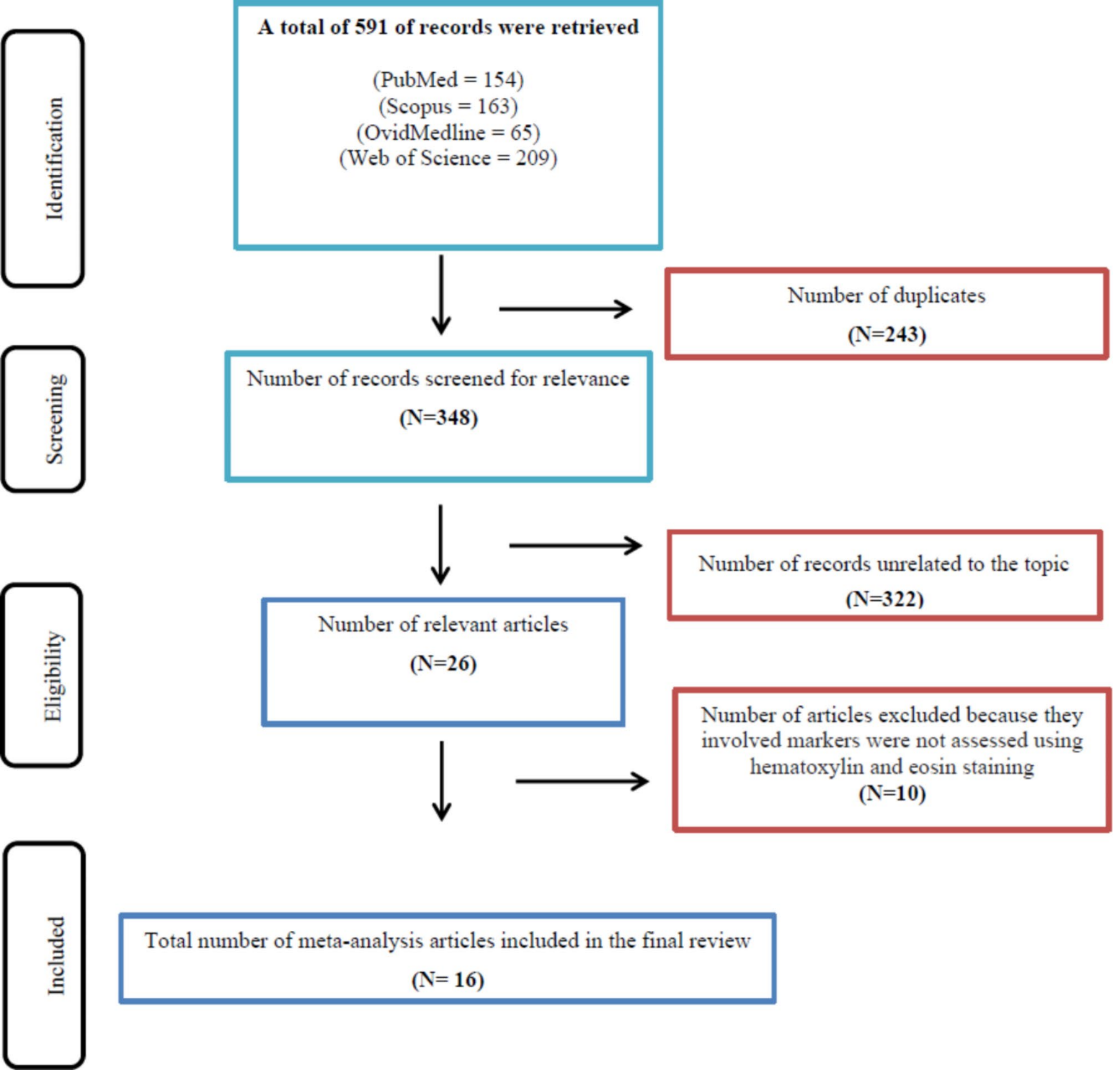
Flow chart shows inclusion and exclusion of articles at each stage of the screening of the retrieved records (adopted from Preferred Reporting Items for Systematic Review and Meta-Analyses, PRISMA). Black boxes: indicate the steps of our systematic search. Blue boxes: indicate the included hits in each stage of the search/filtration. Red boxes: indicate the excluded hits in each stage of the search/filtration

Narrative reviews, cohort studies, case reports, case series. The inclusion and exclusion and reasons for exclusion are illustrated in a PRISMA flowchart (Fig. 1).

### Data extraction

The following information was retrieved from the relevant meta-analyses: First author, year, journal, title of the article, site within oral cavity, histologic marker/s included in the analysis, number of studies, and number of cases in each meta-analysis, survival outcome, prognostic value reported from each meta-analysis and the conclusion of each meta-analysis (Table 1). The hazard ratio, odds ratio, 95% confidence intervals (CIs), and *P*-value were extracted from each meta-analysis whenever reported.

### Risk of bias appraisal

The risk of bias in the included studies was analyzed using the Risk of Bias in Systematic Reviews (ROBIS) tool [15] (Supplementary Table 4).

### Quality appraisal

The quality appraisal of the included systematic reviews was done using the Assessment of Multiple Systematic Reviews (AMSTAR) tool [16] (Supplementary Table 5). The AMSTAR tool has a total of 11 items. Each of the items was assigned a single point. The threshold of the quality of the included studies was set at 50%. Therefore, any studies with less than 50% quality were excluded.

## Results

### Search results

A total of 591 hits were retrieved from database searching. After removal of duplicates and irrelevant articles, 16 meta-analysis articles on histologic prognostic markers assessed in HE-stained sections were eligible to be included. As presented in Table 1, two of the relevant articles included meta-analyses on more than one histologic parameter [17, 18], one article had a meta-analysis on a histopathologic prognostic model [19], while each of the other articles reported meta-analyses on only one parameter.

### Number of included cohorts and prognostic outcomes meta-analyzed

There was a different number of cohorts included in the published meta-analyses with a minimum of two studies and a maximum of 45 studies as presented in Table 1. Lymph node metastasis was analyzed in seven meta-analyses (Table 1). Overall survival was reported in most of the meta-analyses followed by disease-specific survival and disease-free survival (Table 1). The meta-analyses reported hazard ratios or odds ratios (with 95%CI) and, in most cases, also *P-*values (Table 1).

### Summary of findings on histopathologic markers and meta-analyses

There was a total of 11 histopathologic markers/parameters that were included in the published meta-analyses (Table 1). These included meta-analyses for tumor thickness in two articles [17, 20], depth of invasion in two articles [17, 21], perineural invasion in three articles [17, 22, 23], lymphovascular invasion in two articles [17, 24], tumor-associated tissue eosinophilia in one article [25], surgical margins in one article [17], cohesive system in one article [17], worst pattern of invasion in two articles [17, 18], BD model in one article [19], tumor budding in eight articles [7, 17, 18, 26–30] and tumor-stroma ratio in three articles [8, 17, 18]. The BD model, tumor budding and tumor-stroma ratio have been introduced for OSCC only recently, while the other markers were introduced already many years ago. Among the relevant articles, there were a number of meta-analyses that were conducted specifically for studies focusing on early-stage OSCC, and their findings indicated the significance of depth of invasion [21], perineural invasion [23], lymphovascular invasion [24], worst pattern of invasion [18], tumor budding [7, 18, 28] and tumor-stroma ratio [18] in early-stage tumors.

The highest hazard ratio (HR) or odds ratio (OR) of a relationship between a histopathologic prognostic marker and outcome was for depth of invasion and tumors thickness in predicting lymph node metastasis (LNM). The OR for depth of invasion was 10.16 (95% CI 5.05–20.46) based on a meta-analysis of 6 studies (416 patients) as reported by Caldeira et al. [21] and for tumor thickness the OR was 7.32 (95% CI 5.3–10.1) based on a meta-analysis of 16 studies (1136 patients) as reported by Huang SH et al. [20]. In predicting lymph node metastasis, depth of invasion and tumor thickness were followed by lymphovascular invasion with a HR of 5.34 (95% CI 3.44–8.30, *P* < 0.00001) based on a meta-analysis of 18 studies (2161 patients) [24]. The next in prediction of LNM was tumor budding based on a meta-analysis of seven studies (976 patients) with OR of 4.48 (2.97–6.76) as reported by Silva et al. in recent article [29]. For prediction of overall survival, the highest effect estimate was observed for tumor budding with a HR of 3.11 (95% CI 2.06–4.69, *P* < 0.01) as reported by Feitosa et al. [28]; followed by worst pattern of invasion as reported by Dolens et al. [17] with a HR of 2.40 (95% CI 1.19–4.84, *P* = 0.01); then followed by perineural invasion with a HR of 2.36 (95% CI 1.56–3.56, *P* = 0.002) as meta-analyzed by Zhu et al. [22]. In the prediction of disease-specific survival, the highest effect estimate was observed for the cohesive system with HR of 2.63 (95% CI 1.56–4.46, *P* = 0.0003), followed by worst pattern of invasion with a HR of 2.42 (95% CI 1.00-5.88, *P* = 0.05), and then by tumor-stroma ratio with a HR of 2.26 (95% CI 1.65–3.11, *P* < 0.00001) as meta-analyzed by Dolens et al. in one article [17]. For disease-free survival, the highest effect was observed for depth of invasion with OR of 3.83 (95% CI 1.60–9.14, *P* = 0.002) as reported by Caldeira et al. [21], followed by worst pattern of invasion with a HR of 2.82 (95% CI 2.03–3.91, *P* < 0.00001), and then followed by surgical margins with a HR of 2.47 (95% CI 1.90–3.21, *P* < 0.00001) as reported by Dolens et al. [17].

### Risk of bias

Of the 16 studies included in this systematic review, only three studies showed a high risk of bias (Huang 2009 [20]; Zhu J 2019 [22]; & Li 2021 [23]) as presented in Supplementary Table 4. All of the included meta-analyses showed a high quality (over 50%) as shown in Supplementary Table 5.

## Discussion

Biomarker-driven treatment decisions have received valuable research interest recently as they have the potential of aiding in efficient treatment planning. For OSCC, many prognostic biomarkers have been introduced using immunohistochemical staining, RNA sequencing, and other techniques, but, however, they are still in the early stages of development and far away from clinical implementation [31–33]. It has become evident that some histopathologic features are associated with survival in OSCC [6]. Towards a translation into daily practice, many histopathologic markers have been repeatedly validated in their clinical significance, and subsequently, meta-analyses have been conducted [7, 18, 21]. Such an approach can enhance the level of the accumulated evidence and allow to recognize reliable prognostic markers, and to incorporate them in the personalized management of OSCC patients. In this article, we summarized evidence-based knowledge on the clinical significance of histopathologic markers in OSCC in published meta-analyses.

One of the most common and traditional histopathologic parameters in OSCC is the third-dimension measurement, either defined as depth of invasion or as tumor thickness. These have been reported frequently during the last decades and their clinical significance confirmed in many meta-analyses, as summarized in Table 1. For determining tumor thickness and depth of invasion, the usefulness of imaging modalities (including magnetic resonance imaging, computed tomography, and intraoral ultrasonography) and their correlation with histopathologic measurements have been confirmed in recent meta-analyses [34–37]. Of note, depth of invasion has been incorporated in the T class of the most recent edition (i.e. 8th edition) of the TNM classification [38] and this has improved the prognostic performance of the staging system [39].

The invasion of vital structures including nerves, blood and lymphatic vessels in OSCC is a sign of poor prognosis [40] as in other cancers including breast cancer [41] and colorectal cancer [42]. The clinical significance of perineural invasion in OSCC has been reported in three meta-analysis articles [17, 22, 23], while that of lymphovascular invasion has been confirmed by two meta-analyses on OSCC [17, 24] and other cancers [41]. Thus, as the most important histopathologic prognostic parameters perineural invasion and lymphovascular invasion as well as depth of invasion should be considered in clinical decision-making in OSCC. Interestingly, Caponio et al. [43] have recently proposed to incorporate perineural invasion into the TNM staging system of OTSCC. Their proposal still requires further validation.

The histological pattern of tumor invasion in OSCC has been studied since decades using various criteria [44], where the worst pattern of invasion (WPOI) has been one of the most recent. Two meta-analyses have reported that WPOI has reliable prognostic value [17, 18] and accordingly should be taken into account in treatment planning. Of note, tumor budding, defined as single cancer cell/s or cluster/s of less than five cancer cells, has been recently identified as an important prognostic marker in OSCC and various other solid tumors [45]. Tumor budding can be considered a variant histological pattern of invasion with criteria different from those of WPOI, i.e. they overlap but are not identical [46]. However, both tumor budding and WPOI reflect dissociation of tumor growth and the presence of active invasive growth at the tumor front [44]. The clinical significance of tumor budding in OSCC is well-established as presented in eight meta-analyses articles [7, 17, 18, 26–30], and therefore it can advance on the path towards clinical implementation. In risk stratification of tumor budding, a cutoff point of five buds has been widely reported in many studies [7] including a recent comparative study [47]. In addition, a recent meta-analysis has emphasized that five buds is the most often used cutoff point for risk stratification in the published studies [29].

Research on stromal microenvironment of OSCC has been widely conducted using specific staining such as antibodies to alpha-smooth muscle actin [48]. However, this will necessitate additional staining not frequently employed in daily practice. Of note, analysis of tumor-stroma ratio in HE-stained sections of OSCC has been introduced recently and shown promising results in OSCC and other cancers [49–52]. Three meta-analyses [8, 17, 18] have confirmed the prognostic significance of tumor-stroma ratio in OSCC, and this can be a step towards considering the stroma component in pathology reports and clinical decisions. In addition, Mascitti et al. [10] have recently proposed adding tumor-stroma ratio to the TNM staging system of oral tongue cancer and their proposal has shown a promising prognostic value and therefore requires further validation.

Importantly, meta-analyses conducted specifically on studies of early-stage (T1-T2N0M0) OSCC have approved a number of histopathologic markers, namely, tumor budding, tumor-stroma ratio, and worst pattern of invasion, as reliable prognosticators [7, 18]. The standard treatment of early OSCC is surgical resection with or without neck dissection. However, multimodality treatment combining postoperative oncological intervention is sometimes indicated, but this decision can be challenging. In daily practice, this decision is currently based mainly on the pathological TNM staging system, which might not be sufficient as about 18% of early-stage cases are still associated with cancer-related mortality [4]. Thus, the above-mentioned markers in early-stage OSCC can aid in the decision making in such cases. Since many histologic markers have been highlighted as useful prognostic classifiers and they can be assessed in routine HE-stained sections, a multiparameter prognostication approach can ideally be considered using machine learning and web-based tools. This approach for oral cancer has been introduced recently by many researchers [53].

Of note, a few histopathologic markers have been investigated in a small number of studies and therefore, only limited meta-analyses were available for them. For example, the evaluation of eosinophilia has been highlighted in only one meta-analysis article based on a small number of studies with limited number of cases [25]. Similarly, the assessment of tumor-infiltrating lymphocytes (TILs) in HE-stained slides of OSCC has been analyzed in a few studies only [54]. In our recent research including a multicenter study on early oral tongue cancer [55], TILs score has been reported as a powerful prognostic indicator for the assessment of immune response. However, evidence accumulated on the assessment of eosinophilia and TILs in HE-stained slides is still not sufficient to recommend them for daily reporting of OSCC due to the small number of published studies. Thus, more research and validation studies are necessary.

The main limitation/weakness in the published meta-analyses is the heterogeneity of the original cohorts with regard to mixing different oral subsites into the same analysis. It has been reported that SCC of oral subsites (e.g. floor of mouth, oral tongue, gingiva, palate) have different clinical behaviors [56, 57], and therefore, studying each oral subsite separately is recommended. However, each meta-analysis included studies combining different subsites of the oral cavity together. Another heterogeneity in most of the original studies that were meta-analyzed is the combination of early-stage and advanced-stage cases in the same analysis. In addition, another point of weakness is the small number of cases in some original studies of each meta-analysis. Unfortunately, these weaknesses cannot be avoided as they are derived from the original studies. In future research, avoiding such weaknesses is of major importance.

## Conclusions

An overview of the results of the published meta-analyses (Table 1) shows the prognostic impact of histopathologic parameters (that can be assessed in the routine HE-stained sections) including the depth of invasion and perineural invasion. In addition, clinical significance of the recently introduced markers, including tumor budding, worst pattern of invasion and tumor-stroma ratio is confirmed in the pooled analyses of the relevant articles. Of note, in a recent meta-analysis such new markers were also associated with survival in early-stage (T1-T2N0M0) OSCC [18]. Thus, these markers can aid in selecting early-stage OSCC cases that are eligible for a multimodality approach.

Almost all markers highlighted in these meta-analyses are recognized as adverse prognostic factors associated with increased risk for metastasis and a decrease in survival. Due to the use of HE staining in routine histopathology, these markers can be evaluated in the daily practice of pathologists, and could be incorporated in routine pathology reports. In conclusion, based on the existing evidence, we propose the inclusion of the recently introduced markers including tumor budding, worst pattern of invasion, and tumor-stroma ratio in routine histopathological reporting in OSCC, in addition to the older markers, namely, depth of invasion, perineural invasion, and lymphovascular invasion. Although there were some limitations in the above-mentioned published meta-analyses, the level of reliability of the evidence is good as it derives from multiple articles. Thus, these markers can be used for patient stratification to select patients who might benefit from aggressive treatments.

## Electronic supplementary material

Below is the link to the electronic supplementary material.

**Supplementary Material 1**: **Supplementary table 1**: Search strategies for each database.

**Supplementary Material 2**: **Supplementary table 2**: PRISMA checklist for abstract.

**Supplementary Material 3**: **Supplementary table 3**: PRISMA Checklist.

**Supplementary Material 4**: **Supplementary table 4**: Assessment of risk of bias in the included meta-analyses using the Risk of Bias in Systematic Review (ROBIS) tool.

**Supplementary Material 5**: **Supplementary Table 5**: Assessment of the quality of the included studies using modified AMSTAR tool. (AMSTAR: A Measurement Tool to Assess systematic Reviews).

## Data Availability

The datasets used in this study are available from the corresponding author upon a reasonable request.

## Abbreviations

AMSTAR: Assessment of multiple systematic reviews
BD: Budding and depth of invasion combination
CI: Confidence interval
HE: Hematoxylin and eosin
HR: Hazard ratio
LNM: Lymph node metastasis
OR: Odds ratio
OSCC: Oral squamous cell carcinoma
PRISMA: Preferred reporting items for systematic reviews and meta-analyses
ROBIS: Risk of bias in systematic reviews
WPOI: Worst pattern of invasion
