# Supplementary material for: Accumulating evidence from meta-analyses of prognostic studies on oral cancer: towards biomarker-driven patient selection
Source: BMC Cancer. 2024 Dec 18;24:1517. doi: 10.1186/s12885-024-13317-z (PMC11658108; doi:10.1186/s12885-024-13317-z)
Supplement: Supplementary file 1 — Supplementary Material 1: Supplementary table 1: Search strategies for each database. [file 12885_2024_13317_MOESM1_ESM.docx]

**Supplementary Table 1: Search strategies for each database**

| **Database** | **Search strategy** |
| --- | --- |
| PubMed | ("mouth neoplasms"[MeSH Terms] OR ("mouth"[All Fields] AND "neoplasms"[All Fields]) OR "mouth neoplasms"[All Fields] OR ("oral"[All Fields] AND "cancer"[All Fields]) OR "oral cancer"[All Fields]) AND ("meta analysis"[Publication Type] OR "meta analysis as topic"[MeSH Terms] OR "meta analysis"[All Fields]) AND ("marker"[All Fields] OR "markers"[All Fields]) |
| Scopus | TITLE-ABS-KEY ( ( "oral cancer" OR "oral squamous cell carcinoma" ) AND "marker" AND "meta-analysis" ) |
| OvidMedline | (oral cancer.mp. or exp Mouth Neoplasms/ OR oral squamous cell carcinoma.mp. or exp "Squamous Cell Carcinoma of Head and Neck"/) AND (exp Genetic Markers/ or exp Fiducial Markers/ or marker.mp) AND (meta-analysis.mp. or exp Meta-Analysis/) |
| Web of knowledge/web of science | (((ALL=(oral cancer)) OR ALL=(oral squamous cell carcinoma)) AND ALL=(marker)) AND ALL=(meta-analysis) |

**Abbreviation:**

MeSH: Medical Subject Headings
