# Supplementary material for: Accumulating evidence from meta-analyses of prognostic studies on oral cancer: towards biomarker-driven patient selection
Source: BMC Cancer. 2024 Dec 18;24:1517. doi: 10.1186/s12885-024-13317-z (PMC11658108; doi:10.1186/s12885-024-13317-z)
Supplement: Supplementary file 4 — Supplementary Material 4: Supplementary table 4: Assessment of risk of bias in the included meta-analyses using the Risk of Bias in Systematic Review (ROBIS) tool. [file 12885_2024_13317_MOESM4_ESM.docx]

**Supplementary Table 4: Assessment of risk of bias in the included meta-analyses using the Risk of Bias in Systematic Review (ROBIS) tool**

Low risk of bias = $\downarrow$ High risk of bias = $\uparrow$

|  |  | | |  |  |
| --- | --- | --- | --- | --- | --- |
| Articles numbers (Ref: Table 1) | Study eligibility criteria  [Concerns regarding specification of study eligibility criteria] | Identification and selection of studies  [Concerns regarding methods used to identity and/or select studies] | Data collection and study appraisal  [Concerns regarding the methods used to collect data and appraise studies] | Synthesis and findings  [Concerns regarding the synthesis] | Risk of bias in the review |
| 1 | $\uparrow$ | $\uparrow$ | $\downarrow$ | $\downarrow$ | $\uparrow$ |
| 2 | $\downarrow$ | $\downarrow$ | $\downarrow$ | $\downarrow$ | $\downarrow$ |
| 3 | $\downarrow$ | $\downarrow$ | $\downarrow$ | $\downarrow$ | $\downarrow$ |
| 4 | $\uparrow$ | $\downarrow$ | $\downarrow$ | $\downarrow$ | $\uparrow$ |
| 5 | $\downarrow$ | $\downarrow$ | $\downarrow$ | $\downarrow$ | $\downarrow$ |
| 6 | $\downarrow$ | $\downarrow$ | $\downarrow$ | $\downarrow$ | $\downarrow$ |
| 7 | $\downarrow$ | $\downarrow$ | $\downarrow$ | $\downarrow$ | $\downarrow$ |
| 8 | $\downarrow$ | $\downarrow$ | $\downarrow$ | $\downarrow$ | $\downarrow$ |
| 9 | $\downarrow$ | $\downarrow$ | $\downarrow$ | $\downarrow$ | $\downarrow$ |
| 10 | $\uparrow$ | $\downarrow$ | $\downarrow$ | $\downarrow$ | $\uparrow$ |
| 11 | $\downarrow$ | $\downarrow$ | $\downarrow$ | $\downarrow$ | $\downarrow$ |
| 12 | $\downarrow$ | $\downarrow$ | $\downarrow$ | $\downarrow$ | $\downarrow$ |
| 13 | $\downarrow$ | $\downarrow$ | $\downarrow$ | $\downarrow$ | $\downarrow$ |
| 14 | $\downarrow$ | $\downarrow$ | $\downarrow$ | $\downarrow$ | $\downarrow$ |
| 15 | $\downarrow$ | $\downarrow$ | $\downarrow$ | $\downarrow$ | $\downarrow$ |
| 16 | $\downarrow$ | $\downarrow$ | $\downarrow$ | $\downarrow$ | $\downarrow$ |
