# Supplementary material for: Accumulating evidence from meta-analyses of prognostic studies on oral cancer: towards biomarker-driven patient selection
Source: BMC Cancer. 2024 Dec 18;24:1517. doi: 10.1186/s12885-024-13317-z (PMC11658108; doi:10.1186/s12885-024-13317-z)
Supplement: Supplementary file 5 — Supplementary Material 5: Supplementary Table 5: Assessment of the quality of the included studies using modified AMSTAR tool. (AMSTAR: A Measurement Tool to Assess systematic Reviews). [file 12885_2024_13317_MOESM5_ESM.docx]

**Supplementary Table 5: Assessment of the quality of the included studies using modified AMSTAR tool**

AMSTAR: A Measurement Tool to Assess systematic Reviews

Yes: 🗹; No: ⌧

|  |  |  |  | |  |  | | | | | | | | | | | | |
| --- | --- | --- | --- | --- | --- | --- | --- | --- | --- | --- | --- | --- | --- | --- | --- | --- | --- | --- |
| Articles numbers  (Ref: Table 1) | **1** | **2** | **3** | **4** | | | **5** | **6** | **7** | **8** | **9** | **10** | **11** | **12** | **13** | **14** | **15** | **16** |
| AMSTAR Parameters |  |  |  |  | | |  |  |  |  |  |  |  |  |  |  |  |  |
| Was ‘‘a priori’’ design provided? | 🗹 | 🗹 | **🗹** | 🗹 | | | **🗹** | **🗹** | **🗹** | **🗹** | 🗹 | **🗹** | 🗹 | **🗹** | **🗹** | 🗹 | 🗹 | 🗹 |
|  |  |  |  |  | | |  |  |  |  |  |  |  |  |  |  |  |  |
| Was there duplicate study selection and data extraction? | ⌧ | 🗹 | **🗹** | 🗹 | | | **🗹** | **🗹** | **🗹** | **🗹** | 🗹 | **🗹** | 🗹 | **🗹** | **🗹** | 🗹 | 🗹 | 🗹 |
|  |  |  |  |  | | |  |  |  |  |  |  |  |  |  |  |  |  |
| Was a comprehensive literature search performed? | 🗹 | 🗹 | **🗹** | 🗹 | | | **🗹** | **🗹** | **🗹** | **🗹** | 🗹 | **🗹** | 🗹 | **🗹** | **🗹** | 🗹 | 🗹 | 🗹 |
|  |  |  |  |  | | |  |  |  |  |  |  |  |  |  |  |  |  |
| Was the status of publication (i.e., grey literature) used as an inclusion criterion? | ⌧ | 🗹 | **🗹** | 🗹 | | | **🗹** | **🗹** | ⌧ | **🗹** | 🗹 | **🗹** | 🗹 | **🗹** | **🗹** | 🗹 | 🗹 | ⌧ |
|  |  |  |  |  | | |  |  |  |  |  |  |  |  |  |  |  |  |
| Was a list of studies (included and excluded) provided? | 🗹 | 🗹 | **🗹** | 🗹 | | | **🗹** | **🗹** | **🗹** | **🗹** | 🗹 | **🗹** | 🗹 | **🗹** | **🗹** | 🗹 | 🗹 | 🗹 |
|  |  |  |  |  | | |  |  |  |  |  |  |  |  |  |  |  |  |
| Were the characteristics of the included studies provided? | 🗹 | 🗹 | **🗹** | 🗹 | | | **🗹** | **🗹** | **🗹** | **🗹** | 🗹 | **🗹** | 🗹 | **🗹** | **🗹** | 🗹 | 🗹 | 🗹 |
|  |  |  |  |  | | |  |  |  |  |  |  |  |  |  |  |  |  |
| Was the scientific quality of the included studies assessed and documented? | ⌧ | ⌧ | **🗹** | 🗹 | | | **🗹** | **🗹** | **🗹** | **🗹** | 🗹 | **🗹** | 🗹 | **🗹** | **🗹** | 🗹 | 🗹 | 🗹 |
|  |  |  |  |  | | |  |  |  |  |  |  |  |  |  |  |  |  |
| Was the scientific quality of the included studies used appropriately in formulating conclusions? | ⌧ | ⌧ | **⌧** | 🗹 | | | **🗹** | **🗹** | **🗹** | **🗹** | 🗹 | **🗹** | 🗹 | **🗹** | **⌧** | ⌧ | 🗹 | 🗹 |
|  |  |  |  |  | | |  |  |  |  |  |  |  |  |  |  |  |  |
| Were the methods used to combine the findings of studies appropriate? | 🗹 | 🗹 | **🗹** | 🗹 | | | **🗹** | **🗹** | **🗹** | **🗹** | 🗹 | **🗹** | 🗹 | **🗹** | **🗹** | 🗹 | 🗹 | 🗹 |
|  |  |  |  |  | | |  |  |  |  |  |  |  |  |  |  |  |  |
| Was the likelihood of publication bias assessed? | ⌧ | ⌧ | **🗹** | ⌧ | | | **🗹** | **🗹** | **🗹** | **⌧** | ⌧ | **⌧** | 🗹 | **⌧** | **⌧** | 🗹 | 🗹 | 🗹 |
|  |  |  |  |  | | |  |  |  |  |  |  |  |  |  |  |  |  |
| Were potential conflicts of interest included? | 🗹 | 🗹 | **🗹** | 🗹 | | | **🗹** | **🗹** | **🗹** | **🗹** | 🗹 | **🗹** | 🗹 | **🗹** | **🗹** | 🗹 | 🗹 | 🗹 |
|  |  |  |  |  | | |  |  |  |  |  |  |  |  |  |  |  |  |
| Total (%) | 54.5 | 72.7 | 90.9 | 90.9 | | | 100 | 100 | 90.9 | 90.9 | 90.9 | 90.9 | 100 | 90.9 | 81.8 | 90.9 | 100 | 90.9 |
